# Supplementary material for: Hot-Spot-Specific Probe (HSSP) for Rapid and Accurate Detection of KRAS Mutations in Colorectal Cancer
Source: Biosensors (Basel). 2022 Aug 4;12(8):597. doi: 10.3390/bios12080597 (PMC9406089; doi:10.3390/bios12080597)
Supplement: Supplementary file 1 [file biosensors-12-00597-s001.zip › biosensors-1815800 - supplement.pdf]

biosensors

MDPI

Supplementary Information

Hot-Spot-Specific Probe (HSSP) for Rapid and Accurate Detection of KRAS Mutations in Colorectal Cancer

Hyo Joo Lee [1,†], Bonhan Koo [1,†], Yoon Ok Jang [1], Huifang Liu [1], Thuy Nguyen Thi Dao [1], Seok-Byung Lim [2] and Yong Shin [1,*]

1 Department of Biotechnology, College of Life Science and Biotechnology, Yonsei University, 50 Yonsei-ro, Seodaemun-gu, Seoul 03722, Korea; hyoj0125@gmail.com (H.J.L.); bonhan1.koo@gmail.com (B.K.); jangyo17@daum.net (Y.O.J.); liuhuifang.1229@gmail.com (H.L.); ttkh196@gmail.com (T.N.T.D.)
2 Department of Colorectal Cancer, Asan Medical Center, University of Ulsan College of Medicine, 88 Olympic-ro 43-gil, Songpa-gu, Seoul 05505, Korea; sblim@amc.seoul.kr
* Correspondence: shinyongno1@yonsei.ac.kr
† These authors contributed equally to the work.

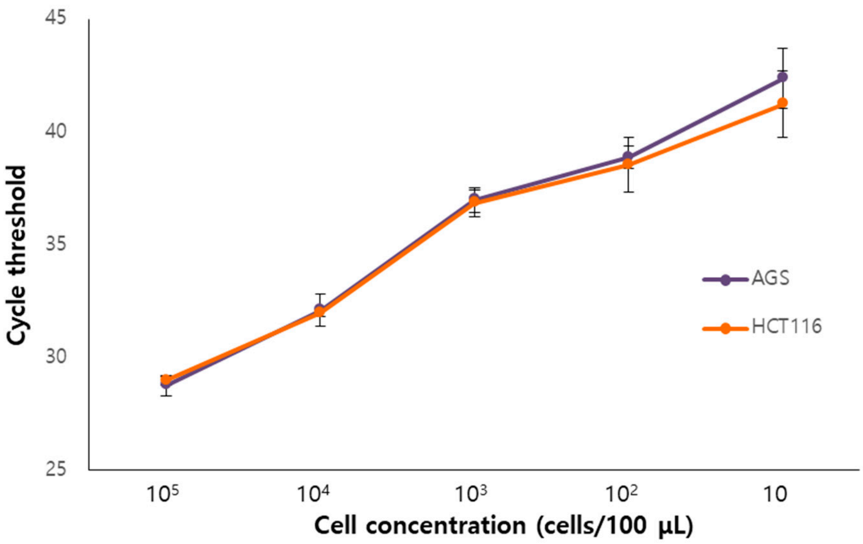

Figure S1. Detection limit of the PCR primer in the cell line. Serial dilution of cell concentration was used to test the detection limit of the PCR primer from cell line extracted DNA. In the same way, up to 10 cells/ 100 μL can be detected in AGS cell line (purple) and HCT116 cell line (orange). Data are presented as mean ± SD, based on at least three independent experiments.

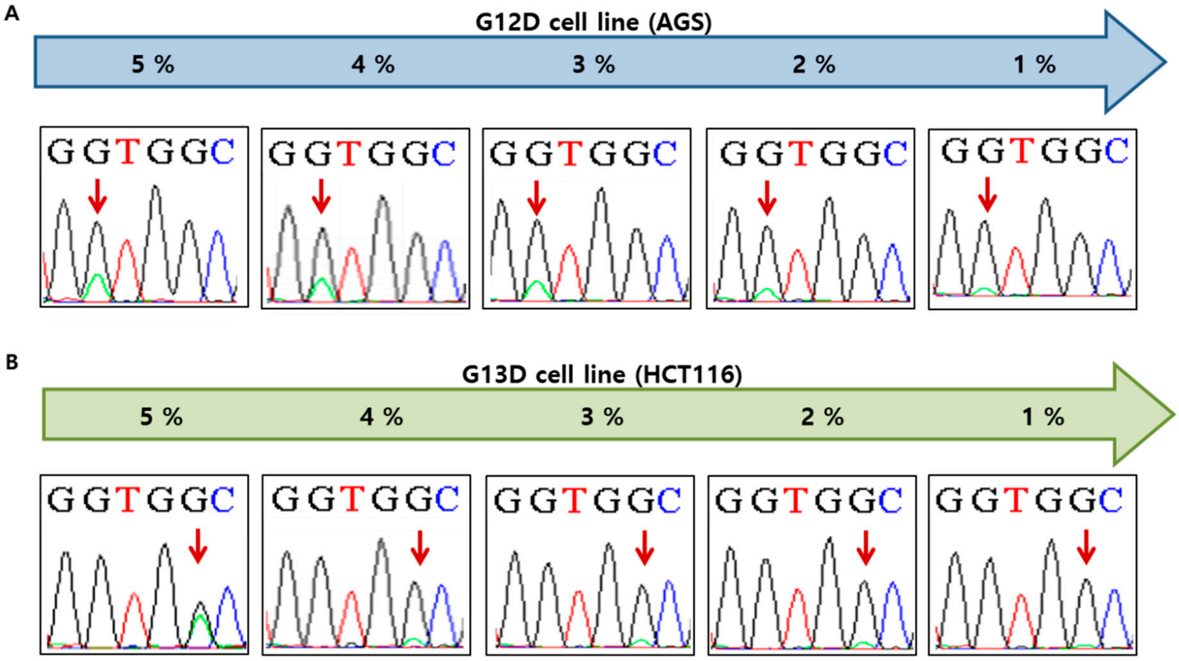

Figure S2. Analysis of the *KRAS* mutations with serially diluted mixed DNA template using HSSP with direct sequencing. (A) *G12D* cell line (AGS) DNA template is by serial dilution with wild-type DNA template. When the AGS DNA template is used at less than 5%, it is possible to detect the mutant DNA templates at less than 1% if additional direct sequencing after qPCR with the HSSP-G13D. (B) *G13D* cell line (HCT116) DNA template is by serial dilution with wild-type DNA template. When the HCT116 DNA template is used at less than 5%, it is possible to detect the mutant template at less than 2% if additional direct sequencing after qPCR with the HSSP-G12D.

Table S1. Sequences of primers and HSSP.

Primer	Sequences (5′–3′)
KRAS 777bp-F	TAGCCGCCGCAGAACAGCAGTC
KRAS 777bp-R	TCACAATACCAAGAAACCCATAAA
KRAS qPCR-F	GCTGTATCGTCAAGGCACTCTT
KRAS qPCR-R	ACCTTATGTGTGACATGTTCTAATATAGTC
HSSP-G12D	GCACTCTTGCCTACGCCA**T**CAGCAAAT - 3′ C3 spacer
HSSP-G13D	GCACTCTTGCCTACG**T**CACCAGCAAAT - 3′ C3 spacer

Table S2. Cancer stage characteristics of CRC patients.

Cancer stage.	Total No. (%)	*KRAS* wild type No. (%)	*KRAS* mutation	
			G12D type No. (%)	*G13D* type No. (%)
**I**	**0 (0%)**	0 (0%)	0 (0%)	0 (0%)
II	4 (5.8%)	3 (15.8%)	2 (8%)	0 (0%)
III	13 (18.8%)	6 (31.6%)	2 (8%)	5 (20%)
IV	52 (75.4%)	10 (52.6%)	21 (84%)	20 (80%)
Total no. of patients	69	19	25	25

Table S3. Differences in the ΔC_t value when using the HSSP in the wild-type DNA template from 10^5 to 10 copies.

Wild-type DNA (copies/reaction)	None	HSSP-G12D	HSSP-G13D	*G12D* − *G13D* (ΔC_t)
10^5	21.373	24.794	23.911	0.882
10^4	26.027	30.832	29.539	1.29
10^3	29.612	34.896	33.465	1.43
10^2	36.295	40.264	39.418	0.84
10^1	39.647	43.293	42.819	0.47

Table S4. Results of HSSP with qPCR, direct sequencing assays in 69 clinical samples.

No.	Type	HSSP-G12D C_t value	HSSP-G13D C_t value	ΔC_t	HSSP with qPCR	Direct Sequenceing
1	G13D	34.951	43.842	-8.891	G13D	G13D
2	wild type	34.246	34.621	-0.375	wild type	wild type
3	wild type	43.55	41.88	1.67	wild type	wild type
4	wild type	29.288	29.662	-0.374	wild type	wild type
5	wild type	26.16	26.011	0.149	wild type	wild type
6	wild type	38.877	41.717	-2.84	G13D	wild type
7	G13D	39.74	45	-5.26	G13D	G13D
8	wild type	27.708	28.367	-0.659	wild type	wild type
9	wild type	28.018	28.448	-0.43	wild type	wild type
10	wild type	25.614	28.658	-3.044	G13D	wild type
11	G13D	37.418	43.14	-5.722	G13D	wild type
12	wild type	40.32	40.529	-0.209	wild type	wild type
13	wild type	35.938	35.536	0.402	wild type	wild type
14	wild type	42.867	42.419	0.448	wild type	wild type
15	wild type	26.421	26.669	-0.248	wild type	wild type

16	G13D	35.144	41.927	-6.783	G13D	G13D
17	wild type	39.523	40.572	-1.049	wild type	wild type
18	G12D	32.787	29.278	3.509	G12D	G12D
19	wild type	26.669	26.748	-0.079	wild type	wild type
20	G13D	25.746	31.316	-5.57	G13D	G13D
21	G13D	26.952	30.026	-3.074	G13D	G13D
22	G12D	43.372	36.108	7.264	G12D	G12D
23	G12D	43.078	37.296	5.782	G12D	wild type
24	G13D	23.564	29.605	-6.041	G13D	G13D
25	G13D	40.205	37.657	2.548	G12D	wild type
26	G12D	45	40.665	4.335	G12D	G12D
27	G12D	37.627	35.067	2.56	G12D	G12D
28	wild type	28.939	28.931	0.008	wild type	wild type
29	G13D	26.004	31.557	-5.553	G13D	G13D
30	G12D	26.226	24.57	1.656	wild type	G12D
31	G12D	35.664	35.683	-0.019	wild type	wild type
32	G13D	34.142	36.51	-2.368	G13D	wild type
33	G13D	35.566	38.007	-2.441	G13D	G13D
34	G12D	33.195	30.551	2.644	G12D	G12D
35	G13D	30.487	35.438	-4.951	G13D	G13D
36	G12D	45	40.63	4.37	G12D	G12D
37	G12D	36.076	32.99	3.086	G12D	G12D
38	G13D	38.22	45	-6.78	G13D	G13D
39	G13D	33.126	36.384	-3.258	G13D	wild type
40	G12D	45	36.7	8.3	G12D	G12D
41	G12D	26.562	22.41	4.152	G12D	G12D
42	wild type	25.736	26.149	-0.413	wild type	wild type
43	G13D	31.814	36.672	-4.858	G13D	G13D
44	wild type	34.118	34.896	-0.778	wild type	wild type
45	G13D	34.14	39.383	-5.243	G13D	G13D
46	wild type	35.581	35.275	0.306	wild type	wild type
47	G12D	26.458	23.79	2.668	G12D	G12D
48	G12D	36.363	31.658	4.705	G12D	G12D
49	G12D	26.18	23.993	2.187	G12D	G12D
50	G12D	37.566	33.281	4.285	G12D	G12D
51	G13D	37.212	40.519	-3.307	G13D	G13D
52	G12D	37.578	31.317	6.261	G12D	G12D
53	G13D	37.301	39.467	-2.166	G13D	G13D
54	G12D	26.537	24.971	1.566	wild type	wild type
55	G13D	33.392	40.286	-6.894	G13D	G13D
56	G12D	36.561	33.957	2.604	G12D	G12D
57	G13D	25.731	29.653	-3.922	G13D	G13D
58	G13D	27.413	27.741	-0.328	wild type	wild type
59	G13D	32.826	36.252	-3.426	G13D	G13D
60	G12D	25.554	23.045	2.509	G12D	G12D
61	G12D	28.848	24.763	4.085	G12D	wild type
62	G13D	33.927	36.787	-2.86	G13D	G13D
63	G12D	27.278	22.861	4.417	G12D	G12D
64	G12D	28.991	25.156	3.835	G12D	G12D
65	G13D	33.803	37.199	-3.396	G13D	G13D
66	G13D	32.557	37.573	-5.016	G13D	G13D
67	wild type	26.313	26.284	0.029	wild type	wild type
68	G12D	33.292	37.477	-4.185	G13D	G13D
69	G12D	34.391	31.592	2.799	G12D	G12D
